# A psychological approach to providing self-management education for people with type 2 diabetes: the Diabetes Manual

**DOI:** 10.1186/1471-2296-7-70

**Published:** 2006-11-27

**Authors:** Jackie Sturt, Hafrun Taylor, Andrea Docherty, Jeremy Dale, Taylor Louise

**Affiliations:** 1 Warwick Medical School, University of Warwick, Coventry, UK; 2 The Heart Manual project, Astley Ainslie Hospital, Edinburgh, UK; 3 Redditch and Bromsgrove Primary Care Trust, Redditch, UK

## Abstract

**Background:**

The objectives of this study were twofold (i) to develop the Diabetes Manual, a self-management educational intervention aimed at improving biomedical and psychosocial outcomes (ii) to produce early phase evidence relating to validity and clinical feasibility to inform future research and systematic reviews.

**Methods:**

Using the UK Medical Research Council's complex intervention framework, the Diabetes Manual and associated self management interventions were developed through pre-clinical, and phase I evaluation phases guided by adult-learning and self-efficacy theories, clinical feasibility and health policy protocols. A qualitative needs assessment and an RCT contributed data to the pre-clinical phase. Phase I incorporated intervention development informed by the pre-clinical phase and a feasibility survey.

**Results:**

The pre-clinical and phase I studies resulted in the production in the Diabetes Manual programme for trial evaluation as delivered within routine primary care consultations.

**Conclusion:**

This complex intervention shows early feasibility and face validity for both diabetes health professionals and people with diabetes. Randomised trial will determine effectiveness against clinical and psychological outcomes. Further study of some component parts, delivered in alternative combinations, is recommended.

## Background

The past decade has seen an international trend towards providing primary care based diabetes services with patient education and self-management at the forefront. The International Diabetes Foundation (IDF) [1] standards advocate that "implementation of diabetes education is learner-centred, facilitates cognitive learning, behaviour change and self-management". These are challenging goals for health care providers to uphold but nonetheless are being incorporated into national health policies. For example, shared decision making is a standard promoted in Finland [2] the Americas [3] and the Netherlands [4]. In the United Kingdom (UK), health professionals are expected to work with people living with diabetes to develop their confidence, skills and knowledge, engage in shared decision making and to provide theory-based structured education [5-7]. It has been proposed that such education should meet four criteria [8]: (i) have a structured, written curriculum (ii) have trained educators (iii) be quality assured, and (iv) be audited.

The relative effectiveness of self-management and patient education interventions for diabetes is varied [9] and the primary outcomes similarly broad with some suggestion that only clinically relevant diabetes end-points (e.g. HbA1c, depression), should be assessed in trials [10]. In a systematic review and meta-analysis of psychological interventions in type 2 diabetes, Ismail et al [11] demonstrated a 0.93% reduction in HbA1c. This suggests the need to further investigate the broad applicability of psychological interventions as a component of self-management programme development.

Systematic reviews of self-management training in asthma [12] and type 2 diabetes [9] conclude that collaborative interventions, where people respond to clinical information and goal setting, represent the most effective approaches for improving clinical outcomes. Systematic reviews have, however, been subjected to some criticism in relation to the assessment of randomised controlled trials (RCT) of complex interventions [13-15]. Reviewers themselves also report the difficulties in assessing the quality of trials of complex interventions [12,16,17]. Of fundamental concern is the absence of adequate specification of intervention components in published trials to allow reliable inclusion in systematic reviews and to facilitate effective clinical appraisal of the intervention.

**Figure 1:**
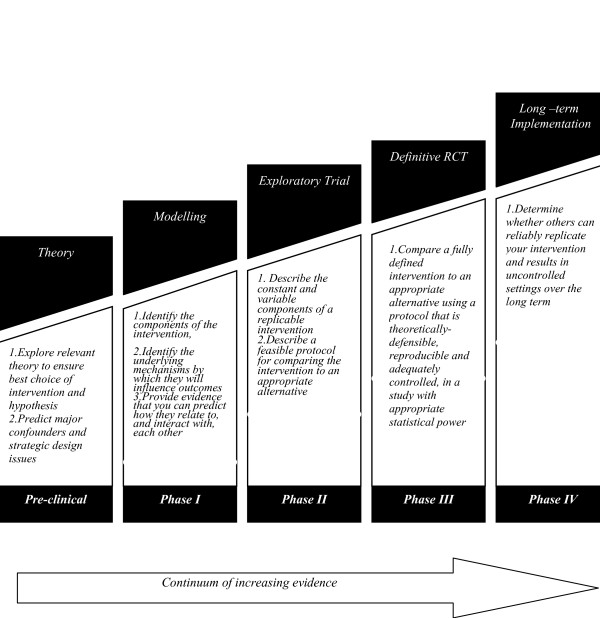
MRC Framework of complex interventions.

Self-management interventions are intrinsically complex [14,18,19] and include a range of organisational (e.g. consultation length) and individual (e.g. nurse training) components. Within a complex intervention, the "active" component may be difficult to describe. The UK's Medical Research Council (MRC) framework for the development and evaluation of complex interventions [20] (figure 1) provides a structure through which the variable components may be tested and compared, both theoretically (pre-clinical phase) and clinically (phase I/II). The framework proceeds towards the development of a phase III/IV trial in which the intervention is subjected to RCT and long-term, pragmatic studies. Publication of the preliminary phases can provide theoretical and feasibility detail and specification of intervention components which can begin to address concerns [13,14]. This paper reports the early phase development and evaluation of a complex intervention.

## Methods

### Research aims

The broad research aims were to develop the Diabetes Manual, a type 2 diabetes self-management educational intervention, and associated programme for delivery in primary care. The Diabetes Manual was required to meet the UK policy standards for diabetes care [5-8], and those of the IDF [1] using intervention development and evaluation methodology offered by the MRC framework [20]. Specific development objectives were i) to use self-efficacy theory to inform the structure and process of the Diabetes Manual programme in order to influence the behaviour of health care professionals and people with diabetes? ii) to address the self-management needs of people with type 2 diabetes within the proposed structure iii) to test acceptability and face validity of the Diabetes Manual format among members of the target population and primary care professionals. This work aims to inform subsequent phase II-IV trials and systematic reviews of effectiveness.

### Pre-clinical (theoretical) phase

#### Theory

The theoretical basis for the Diabetes Manual is Bandura's [21] self-efficacy theory. Self-efficacy is empirically recognised as one of the strongest predictors of health behaviour change [22-24] and is defined as an individual's level of confidence in their ability to perform a particular behaviour (efficacy expectations). Randomised trials of interventions that incorporate specific efficacy-enhancing techniques of (i) experiencing personal mastery, (ii) positive vicarious learning, (iii) adjustment to stress and (iv) positive verbal persuasion have demonstrated fewer episodes of hospitalisation and improved psychosocial adjustment to a new health status [18] and reduction in body mass index and HbA1c [25]. Self-efficacy can be measured using validated scales specific to the behaviours and activities at which an intervention aims to influence [26-28].

#### Needs assessment

Evidence to inform the design of the Diabetes Manual emerged from a patient self-management education needs assessment study [29] undertaken in 2002. This 23 participant focus group study of people with a new diagnosis or a new change in therapy concluded that people with diabetes want educational inputs that incorporate (i) the provision of information (e.g. relationship between physical activity and blood glucose (BG) levels), (ii) training in personal monitoring (length of walk and any resulting reduction in BG level), and skill development in (iii) specific (making time to walk) and (iv) general behavioural goal setting and evaluation (recording progress). This research, whilst limited is its size, identified the needs of people with type 2 diabetes from the target population for whom the Diabetes Manual was intended. These included people with a recent diagnosis and a considerable informational need alongside those with established self-management patterns who might benefit from a refresher course.

#### The Heart Manual

The approach taken by the Heart Manual, an exemplar of good practice [5], provided the strongest piece of pre-clinical evidence for the design of the Diabetes Manual. The Heart Manual (HM) includes all the elements of a comprehensive rehabilitation programme for coronary heart disease (CHD) including information, goal setting and evaluation, exercise initiation, stress management and relaxation, smoking cessation, nutrition and weight loss, cholesterol reduction, medication, prognosis, patient vignettes and attention to psychological needs. The programme is delivered by trained facilitators, usually nurses, and consists of a 6-week workbook, 2 audio-tapes and 3 facilitator telephone support calls.

The HM is underpinned by cognitive behaviour therapy and was developed and evaluated following a trajectory loosely in line with the MRC framework. Over a one-year period, individuals and small groups of patients were presented with sections to work through at home. Following completion of each section, patients were interviewed to determine where adherence was stronger/weaker and to identify the most useful components. Based on this feedback each section was repeatedly rewritten. The first phase III randomised trial of the HM [30] was carried out with 176 post-MI patients randomised to receive the HM programme or follow a control group protocol. The control group received phone calls at 1, 3, and 6 weeks post-MI, plus information leaflets on recovery from MI and lifestyle changes. The study measured anxiety and depression, confidence, quality of life, contact with GP, hospital re-admissions and self rating on recovery. Follow-up data were obtained for 109 participants. The intervention group demonstrated significant reductions in anxiety (p => 0.04), GP/hospital contacts (p =< 0.05), clinical anxiety (p =< 0.001) and depression (p =< 0.03). The presence of depression in those who have experienced a myocardial infarction reduces return to functionality [31]. People with diabetes are twice as likely to have clinical depression as the non-diabetic population [32] and depression is associated with reduced engagement in self-management activities [33] and poorer glycaemic control [34]. Therefore a self-management intervention which may reduce anxiety and depression has clear clinical relevance in diabetes. Several phase IV pragmatic trials of the HM have been undertaken measuring effectiveness across a range of biomedical and psychosocial outcomes [35-38].

In addition to presenting the theoretical foundations and assessment of previous empirical study, this phase should consider strategic design issues and major confounders. The intervention idea was consistent with current diabetes health policy [1,5-8] and therefore strategically secure. The role of this same health policy was to result in an unpredicted confounder for the ongoing RCT and has been described elsewhere [39].

In summary, the Heart Manual evidence base was strong [30,38]. Empirical evidence indicated the strong predictive value of self-efficacy for informing targeted goal setting and evaluation which were amongst the self-management educational needs identified by people with type 2 diabetes. We hypothesized from the pre-clinical work that a Diabetes Manual, designed and delivered to enhance diabetes self-management self-efficacy, would be feasible to people with diabetes at differing time points from diagnosis and to health care professionals.

## The Diabetes Manual phase I (modelling) study

### Feasibility work

Ethical permission was obtained in 2002 to determine the views of health care professionals and people with type 2 diabetes on self-management interventions [40]. Eight diabetes professionals participated in semi-structured interviews which revealed support for the Diabetes Manual approach and cautioned on the need for interventions which do not promote overdependence on professionals. A questionnaire focusing upon areas of difficulty and need were mailed to 300 people with type 2 diabetes from three General Practices in Coventry and Warwickshire, UK. Eighty-five (28%) people completed the questionnaire, of whom 47 (55%) were female, mean age was 63 years (range 19 to 90 years) and 96.4% considered themselves white, white British or European. The mean length of diagnosis was 9 years (range 1 month to 52 years). In relation to current treatment, 5% were managing through diet and exercise, 71% were taking oral medication, and 24% were using insulin injections. 94% of respondents considered the description of the Diabetes Manual approach to be useful and indicated their preference for content (table 1). The suggested content was adapted from the Heart Manual workbook and this survey found that the content and structure of a hypothetical Diabetes Manual demonstrated feasibility, in principle, with its intended population of people with type 2 diabetes and primary care professionals.

**Table 1:** Preferences of people with diabetes for intervention content

**Suggested Item**	**Percentage of respondents indicating preference for item inclusion**
Most commonly asked questions	88%
Explanation – what to expect	81%
Exercise programme	61%
Diet information	87%
Information on medication	72%
In case of emergency	87%
Advice about risk	74%
Advice on changing lifestyle	55%
Relaxation and stress management	59%
Learning about others experiences	55%
Tape cassette for family	34%

### Workbook development

This phase aimed at ensuring that the intended efficacy-enhancing components (e.g. positive mastery experiences) were clearly incorporated into the workbook as this is the mechanism through which we aim to influence diabetes outcomes. In 2003, two development panels were convened; the first was a 7-member lay panel of people living with diabetes. The second was a 13-member diabetes health professional panel, which included a nurse, a dietician, diabetes consultants, GPs, researchers, a health psychologist, a podiatrist and an exercise consultant. South Asian, Afro Caribbean and white English ethnicity groups were represented and the panels advised on the content and design of the workbook. Both panels met individually on 4 occasions with the workbook writer, a HM board member, over a 13-month period. During this time, panel members were charged with reviewing content, personally, and within their own diabetes networks. Using the pre-clinical and the phase I evidence [29,38,40] and diabetes policy targets [6], the core syllabus was agreed and written, and structure determined. It was a unanimous view that the workbook should be a 12 week programme as opposed to the HM's six, reflecting the relative differences between rehabilitation following an acute myocardial infarct and imposed hospitalisation and learning to live with diabetes. To establish face validity 12 people with diabetes, who had no previous involvement in the workbook, read it over a 2-week period and attended a focus group discussion. Unprompted, each participant volunteered personal lifestyle changes they had decided to make as a consequence of reading the draft. Further design changes were considered by the development panels as a consequence of participants' feedback.

### Audiotapes

Stress management and anxiety reduction was determined to be important by the Diabetes Manual lay panel. Reinterpretation of physiological response to emotional distress is a core efficacy enhancing mechanism and the relaxation audio-tapes worked with the workbook text to focus on these messages and offered a therapeutic solution to reduce anxiety. A generic relaxation tape, recorded by the HM team, was determined by the lay group to be suitable for use with a diabetes population. The "frequently asked questions" tape script was drafted by the lay group and refined further using the empowerment model approach [41] to communication. This provided positive vicarious experiences of the types of discussions it is possible, and permissible, for patients to hold with health care professionals.

### Telephone support

Systematic review of the telesupport in healthcare literature [42] has concluded that telecommunication for patient care is acceptable to patients. Automated telephone diabetes management has been shown to reduce depressive symptoms and increase self-efficacy in comparison with usual care [43]. Our phase I feasibility study [40] revealed that 83% (n = 71) of respondents would prefer telephone support from a known health professional. This suggested that the practice nurse might be the most appropriate telephone supporter due to their pre-existing relationship with the patient. It also carried the potential for the intervention effect to be lengthened as the practice nurse would have skills available for use during post-intervention routine diabetes reviews. Telephone support enables the patient to develop mastery through reflecting on their successes with an informed and trusted individual who can also offer verbal encouragement.

Nurse training – To use self-efficacy theory to approach patient and professional behaviour change, goal setting, goal achievement planning and evaluation should to occur. This has similarity to experiential learning described by Lewin [44] and Kolb [45]. As a social learning theory, self-efficacy is modified by social stimuli provided vicariously and is therefore appropriate as a theoretical framework for curriculum design for use in group teaching. The objective of the small group nurse training was to provide a 2-day event during which time the nurses increased their understanding, skills and confidence for delivering the Diabetes Manual. As the nurses need to deliver the intervention in accordance with the principles and mechanisms of self-efficacy theory, written materials were developed that had demonstrated effectiveness in earlier phase I studies [46-48]. The curriculum was developed with regard to promoting the nurses self-efficacy for delivering the intervention. Telephone support, for example, is a new therapeutic arena for the delivery of care. The nurses engaged in role play to develop their own skills and observe those of others. In this way their self-efficacy for delivering telephone care was enhanced through vicarious experiences and their own personal mastery along with verbal encouragement from the nurse trainer and discussion, and reinterpretation, of their own telephone anxiety.

The Diabetes Manual for people living with type 2 diabetes was completed in April 2004.

## Results

Our research questions were i) How could self-efficacy theory inform the structure and process of the Diabetes Manual programme in order to influence the behaviour of health care professionals and people with diabetes? ii) How could the self-management needs of people with type 2 diabetes be addressed within the proposed structure? iii) Would the proposed Diabetes Manual format have face validity for people with the target population and primary care professionals?

The workbook and component development work took the content listed in table 1, along with the original Heart Manual, and established a Diabetes Manual intervention incorporating some existing content, new original diabetes material and an efficacy enhancing structure designed to weave throughout the workbook and the programme as a whole. This data is presented in table 2 and resulted in the production of the Diabetes Manual intervention. The Diabetes Manual consists of a 2-day training event for practice nurses, a 230 page workbook for recommended completion over a 12 week period, 2 audio tapes and a telephone support component. It is delivered by primary care nurses in a single 15 minute face to face consultation to individuals with a new diagnosis of type 2 diabetes or the recognition of sub-optimal diabetes control (i.e. a raised HbA1c). The patient takes the workbook and audio-tapes home to commence the programme following the consultation and receives follow-up support during 10-minute telephone calls in weeks 1, 5 and 11. The workbook is presented as a staged process within which the patient moves naturally from one section to the next with each section gradually building on knowledge and experience gained in the earlier sections. Each section includes core content, such as choices and encouragement regarding physical activity, nutrition and home blood glucose monitoring. A new aspect of diabetes management is incorporated in amongst the earlier, more fundamental content by focussing on cholesterol, blood pressure, smoking or stress as the weeks progress. As the majority of the intervention is patient directed, patients can choose where they turn their attention and the telephone support is provided to help patients to evaluate their chosen goals and maintain enthusiasm rather to redirect them to sequential activity. The relationship of each component to the theoretical basis is presented alongside the knowledge and coaching syllabus in table 2 to map how the components enhance efficacy and outcome expectations.

**Table 2:** Diabetes Manual components and mechanisms through which self-efficacy is influenced

**Diabetes Manual programme component**	**Knowledge and coaching syllabus**	**Self-efficacy enhancement**
2-day training for practice nurses (PN) experienced in diabetes management.	Self-efficacy theory and adult learning. Intervention structure Practical skill development in telephone support and empowering communication	Mastery achievements Positive vicarious learning Adjustment to stress Verbal encouragement Outcome expectations
Patient workbook approached over 12 weeks	Diabetes facts /Metabolism /Goal setting and evaluation /Exercise /Nutrition/Blood glucose monitoring /Weight loss /Smoking cessation /tests /Complications /Medication /Vignettes /Stress, anxiety and depression /Cholesterol.	Mastery achievements Vicarious experiences
Relaxation audiotape	Teach techniques and facilitate opportunities	Adjustment to stress
Question and answer audiotape	Provides for quick diabetes self-management recall for patient and carers/family	Promotes mastery achievements Vicarious learning
PN telephone support	Assess goal progress; patient recollection of goal achievement, promotion of goal self- evaluation and re-negotiation.	Mastery experiences Verbal encouragement

Strategic design criteria [8], published near the point at which the Diabetes Manual was completed, further justifies its development and retrospectively contributes pre-clinical data. Four criteria against which structured education for people with diabetes should be judged in the UK is presented in table 3, providing evidence to indicate where, and how, the Diabetes Manual meets these criteria.

**Table 3:** Criteria for UK Structured Education Programmes

**Criteria 1 " A structured curriculum" needs to be......**	**Where the Diabetes Manual meets the criteria**
1)Person centred, incorporating individual needs assessment	• Initiated according to individual assessment. • Patient prioritises sequences of activity
2)Reliable, valid and comprehensive	• Evidence and policy based. • Stakeholder input to development
3)Theory driven	• Self-efficacy and experiential learning theory
4)Flexible and available to diverse groups	• Self-managed by individual, 1 hour per day including exercise regimen, limited contact time with nurse negotiated. • Reading age of 12 years. • Culturally sensitive, e.g. Vignettes, cartoons, nutrition.
5)Use different teaching medium	• Text, pictures, personal reflection and evaluation, audiotape, one to one contact with nurse
6)Resource effective	• 45 mins additional nurse contact time. • No economic evaluation of DM. HM currently costs €20–32 per person treated to purchase incl training and tapes.
7)Written down	• 230 page workbook

**Criteria 2 "Trained educators" need to.......**	**Where the DM training meets the criteria**

1)Understand education theory as relevant to particular learners	• Taught principles of self-efficacy theory and experiential learning related to individual patient learning needs. • Role play experiences teaching by theses methods.
2)Be trained and competent in the delivery of the education theory	• Role play and rehearsal of face to face and telephone consultation using empowering and efficacy enhancing communications
3)Be trained and competent in the delivery of the principles and content of the specific programme they are offering	• Diabetes Manual workbook is evidence based. • Material used as a reference source for nurse if required. • Skill development in working through the stages

## Discussion

The Diabetes Manual is modelled on the Heart Manual and it is clear that the contexts for their respective use vary considerably. Contextual differences have been addressed through significant development of the Diabetes Manual but the degree to which a programme will transfer from an acute episode to a chronic condition remains to be evaluated in an ongoing RCT. The decision to extend the programme from six to 12 weeks reflects this contextual uncertainty. The Heart Manual was designed to promote psychological adjustment to CHD and measured no biomedical outcomes. The Diabetes Manual has been designed to promote increased self-efficacy for diabetes self-management and in consequence, improve HbA1c and CHD risk factors, along with self-efficacy [49] and quality of life [50]. The Diabetes Manual phase I study has invested in the effectiveness of the Heart Manual in its entirety. It is, therefore, less clear whether all the component parts of the Diabetes Manual are necessary.

Further study limitations lie in the limited exposure the Diabetes Manual has had to non-self selecting populations. The phase I primary care feasibility survey [40] which preceded the workbook development had a response rate of only 28%. Further exposure of the Diabetes Manual to people with diabetes, through the lay development groups and the focus groups who read the penultimate draft, has been through contacts initiated via the diabetes voluntary sector. The literacy-based nature of a large part of the intervention may limit its comprehensive use by groups with poor literacy skills. It has a reading age commensurate with that of the popular British tabloid newspapers.

The development process was theoretically rigorous, involved multiple stakeholders and the Diabetes Manual programme demonstrates strong face validity with both professionals and people with diabetes.

## Conclusion

This paper provides evidence for the validity of the development of the Diabetes Manual according to the principles of the MRC framework [20]. In its use of teaching methods for both practice nurses and people with diabetes, it is learner-centred and facilitates cognitive learning and behaviour change [1]. The programme uses self-efficacy theory [21] and experiential learning techniques [45] to develop confidence, skills and knowledge in diabetes self-management, promote shared decision making with the diabetes team and provide theory-based structured education [5-7]. It has a written curriculum and trained educators [8].

Early publication of this complex intervention development and evaluation data will enable clinicians and future systematic reviewers to determine the specification and evidence base for intervention components. The science of complex intervention development is in its infancy and intervention modelling pathways are emerging to inform this activity [51]. The MRC framework provided a useful structure through which to examine our theoretical hypothesis and analyse the feasibility evidence. The Diabetes Manual is currently being evaluated by randomised controlled trial in the UK to test its effectiveness on HbA1c, BP, BMI, total cholesterol and HDL, self-efficacy and quality of life. A Dutch translation of the entire intervention is undergoing evaluation by quasi-randomised trial in the Netherlands. An ongoing phase III RCT trial will determine its effectiveness for improving both clinical and psychological outcomes for people with diabetes and test quality assurance and audit procedures for its delivery. Further research should seek to understand in greater depth, the relationship between the many constituent parts of this intervention for people with diabetes.

## Competing interests

The authors declare that they have no competing interests.

## Authors' contributions

JS conceived the development of the intervention. HT and LT provided the Heart Manual evidence. JS lead the development of the intervention. JS, JD and AD undertook feasibility work.

## Pre-publication history

The pre-publication history for this paper can be accessed here:


